# Position paper on screening for breast cancer by the European Society of Breast Imaging (EUSOBI) and 30 national breast radiology bodies from Austria, Belgium, Bosnia and Herzegovina, Bulgaria, Croatia, Czech Republic, Denmark, Estonia, Finland, France, Germany, Greece, Hungary, Iceland, Ireland, Italy, Israel, Lithuania, Moldova, The Netherlands, Norway, Poland, Portugal, Romania, Serbia, Slovakia, Spain, Sweden, Switzerland and Turkey

**DOI:** 10.1007/s00330-016-4612-z

**Published:** 2016-11-02

**Authors:** Francesco Sardanelli, Hildegunn S. Aase, Marina Álvarez, Edward Azavedo, Henk J. Baarslag, Corinne Balleyguier, Pascal A. Baltzer, Vanesa Beslagic, Ulrich Bick, Dragana Bogdanovic-Stojanovic, Ruta Briediene, Boris Brkljacic, Julia Camps Herrero, Catherine Colin, Eleanor Cornford, Jan Danes, Gérard de Geer, Gul Esen, Andrew Evans, Michael H. Fuchsjaeger, Fiona J. Gilbert, Oswald Graf, Gormlaith Hargaden, Thomas H. Helbich, Sylvia H. Heywang-Köbrunner, Valentin Ivanov, Ásbjörn Jónsson, Christiane K. Kuhl, Eugenia C. Lisencu, Elzbieta Luczynska, Ritse M. Mann, Jose C. Marques, Laura Martincich, Margarete Mortier, Markus Müller-Schimpfle, Katalin Ormandi, Pietro Panizza, Federica Pediconi, Ruud M. Pijnappel, Katja Pinker, Tarja Rissanen, Natalia Rotaru, Gianni Saguatti, Tamar Sella, Jana Slobodníková, Maret Talk, Patrice Taourel, Rubina M. Trimboli, Ilse Vejborg, Athina Vourtsis, Gabor Forrai

**Affiliations:** 1 Department of Biomedical Sciences for Health, University of Milan, Milan, Italy; 2 Department of Radiology, Research Hospital Policlinico San Donato, Via Morandi 30, 20097 San Donato Milanese, Milan Italy; 3 Department of Radiology, Haukeland University Hospital, Jonas Lies vei 65, 5021 Bergen, Norway; 4 Department of Radiology, Reina Sofía Hospital, Avda. Menéndez Pidal, s/n 14004 Córdoba, Spain; 5 Department of Radiology, Karolinska University Hospital, Karolinska vägen, 171 76 Solna, Stockholm Sweden; 6 Department of Radiology, Meander Medical Center, Maatweg 3, 3813 Amersfoort, The Netherlands; 7 Department of Radiology, Gustave-Roussy Institute, 114 Rue Edouard Vaillant, 94800 Villejuif, France; 8 Department of Biomedical Imaging and Image-guided Therapy, Division of Molecular and Gender Imaging, Medical University of Vienna/Vienna General Hospital, Währinger Gürtel 18-20, 1090 Wien, Austria; 9 Clinic of Radiology, University Clinical Center Sarajevo, Bolnička 25, 71000 Sarajevo, Bosnia and Herzegovina; 10 Clinic of Radiology, Charité, Universitätsmedizin Berlin, 10117 Berlin, Germany; 11 Diagnostic Imaging Center, Institute of Oncology, Institutski put 4, 21204 Sremska Kamenica, Serbia; 12 Radiology Department, National Cancer Institute, Santariskiu 1, Vilnius University, Vilnius, Lithuania; 13 Department of Diagnostic and Interventional Radiology, University Hospital ‘Dubrava’, University of Zagreb School of Medicine, Avenija Gojka Šuška 6, 10000 Zagreb, Croatia; 14 Department of Radiology, Hospital de la Ribera, Carretera de Corbera, Km. 1, 46600 Alzira, Valencia Spain; 15 Radiology Unit, Hospices Civils de Lyon, Centre Hospitalo-Universitaire Lyon Sud, 165 Chemin du Grand Revoyet, 69495 Pierre Bénite Cedex, France; 16 Nottingham Breast Institute, Nottingham University Hospitals, Hucknall Rd, Nottingham, NG5 1PB United Kingdom; 17 Department of Radiology, Charles University in Prague, First Faculty of Medicine, U Nemocnice 2, 128 08 Praha 2, Czech Republic; 18 Department of Radiology, ImageRive, Rue de Rive 1, 1204 Genève, Switzerland; 19 Department of Radiology, Acibadem University Maslak Hospital, Buyukdere cd, Maslak, Sariyer, 34457 İstanbul, Turkey; 20 Dundee Cancer Centre, Clinical Research Centre, Ninewells Hospital, and Medical School, Tom McDonald Avenue, Dundee, UK; 21 Division of General Radiology, Department of Radiology, Medical University Graz, Auenbruggerplatz 9, 8036 Graz, Austria; 22 Department of Radiology, University of Cambridge, Cambridge Biomedical Campus, Hills Road CB2 0QQ, Cambridge, UK; 23 Institute of Radiology, ACC Steyr, Stadtplatz 30, 4400 Steyr, Austria; 24 Department of Radiology, Mater Misericordiae University Hospital/BreastCheck, BreastCheck, 36 Eccles St, Dublin 7, Ireland; 25 Referenzzentrum Mammographie Munchen, Sonnenstraße 29, 80331 Munich, Germany; 26 Diagnostic Department of Radiology, Tokuda Hospital Sofia, 1407 Sofia, 51B N Vaptsarov Blvd., Bulgaria; 27 Department of Radiology, Landspitali University Hospital, 108 Reykjavik, Iceland; 28 University Hospital of Aachen, Rheinisch-Westfälische Technische Hochschule, Pauwelsstraße 30, 52074 Aachen, Germany; 29 Radiotherapy I, The Oncology Institute ‘Prof. Dr. Ion Chiricuta’, Cluj-Napoca, 34-36 Republicii Street, RO-400015 Romania; 30 Department of Radiology Maria Sklodowska-Curie Memorial Cancer Centre and Institute of Oncology, Cracow Branch, Cracow, Poland; 31 Department of Radiology, Radboud University Nijmegen Medical Centre, Geert Grooteplein Zuid 10, 6525 GA Nijmegen, The Netherlands; 32 Department of Radiology/Breast Imaging, Instituto Português de Oncologia de Lisboa, Rua Professor Lima Basto, 1099-023 Lisboa, Portugal; 33 U.O. Radiodiagnostica, Candiolo Cancer Institute - FPO, IRCCS, Str. Prov. 142, km 3.95, 10060, Candiolo, Turin, Italy; 34 Genitourinary Radiology, Ghent University Hospital, De Pintelaan 185, 9000 Gent, Belgium; 35 Klinik für Radiologie, Neuroradiologie & Nuklearmedizin, Klinikum Frankfurt Hoechst, Frankfurt Germany; 36 Diagnoscan, SZTE Radiológiai Klinika, Szeged, Semmelweis u. 6, 6725 Szeged, Hungary; 37 Breast Imaging Unit, Scientific Institute (IRCCS) Ospedale San Raffaele, Via Olgettina, 60, 20132 Milan, Italy; 38 Department of Radiological, Oncological and Pathological Sciences, Sapienza University, Viale Regina Elena, 324, 00161 Rome, Italy; 39 Department of Imaging, University Medical Centre Utrecht, Heidelberglaan 100, 3584 CX Utrecht, The Netherlands; 40 Department of Diagnostic Radiology, Oulu University Hospital, Kajaanintie 50, 90220 Oulu, Finland; 41 Department of Radiology and Medical Imaging, State University of Medicine and Pharmacy ‘Nicolae Testemitanu’, bul. St.cel Mare 165, Chisinau, MD 2012 Moldova; 42 Italian Group for Mammographic Screening (GISMa); Senology Unit, Local Health Authority, Bologna, Italy; 43 Marlene Greenebaum Diagnostic Breast Center, Department of Radiology, Hadassah Hebrew University Hospital, Hadassah Ein Kerem Medical Center, 91120 Jerusalem, Israel; 44 Clinic of Radiology, Alexander Dubček University of Trenčín in Trenčín, Faculty of Healthcare, Študentská 2, 911 50, Trenčín, Slovakia; 45 Radiology Centre, North Estonia Medical Centre, 19 Sütiste Street, 13419 Tallinn, Estonia; 46 Department of Radiology, Hôpital Lapeyronie, 371 Av. du Doyen Gaston Giraud, 34295 Montpellier, France; 47 Department of Radiology, Rigshospitalet Blegdamsvej and Glostrup, Mammography Screening Programme in Capital Region, Blegdamsvej 9, 2100 Copenhagen, Denmark; 48 Diagnostic Mammography Center, Kifisias 362, Chalandri, 15233 Athens Greece; 49 Duna Medical Center, Lechner Ödön fasor 7, 1095 Budapest, Hungary

**Keywords:** Breast cancer, Population-based screening, Digital mammography, Digital breast tomosynthesis (DBT), Recall rate

## Abstract

**Abstract:**

EUSOBI and 30 national breast radiology bodies support mammography for population-based screening, demonstrated to reduce breast cancer (BC) mortality and treatment impact. According to the International Agency for Research on Cancer, the reduction in mortality is 40 % for women aged 50–69 years taking up the invitation while the probability of false-positive needle biopsy is <1 % per round and overdiagnosis is only 1–10 % for a 20-year screening. Mortality reduction was also observed for the age groups 40–49 years and 70–74 years, although with “limited evidence”. Thus, we firstly recommend biennial screening mammography for average-risk women aged 50–69 years; extension up to 73 or 75 years, biennially, is a second priority, from 40–45 to 49 years, annually, a third priority. Screening with thermography or other optical tools as alternatives to mammography is discouraged. Preference should be given to population screening programmes on a territorial basis, with double reading. Adoption of digital mammography (not film-screen or phosphor-plate computer radiography) is a priority, which also improves sensitivity in dense breasts. Radiologists qualified as screening readers should be involved in programmes. Digital breast tomosynthesis is also set to become “routine mammography” in the screening setting in the next future. Dedicated pathways for high-risk women offering breast MRI according to national or international guidelines and recommendations are encouraged.

***Key points*:**

• *EUSOBI and 30 national breast radiology bodies support screening mammography*.

• *A first priority is double*-*reading biennial mammography for women aged 50*–*69 years*.

• *Extension to 73*–*75 and from 40*–*45 to 49 years is also encouraged*.

• *Digital mammography* (*not film*-*screen or computer radiography*) *should be used*.

• *DBT is set to become* “*routine mammography*” *in the screening setting in the next future*.

## Introduction

This position paper on screening for breast cancer (BC) has been proposed by the Executive Board and the Scientific Committee of the European Society of Breast Imaging (EUSOBI) and approved by 30 national breast radiology bodies/sections (Table 1). The aim is to give a clear message in favour of screening mammography to national/local governments, policy makers, referring physicians and the general population.

**Table 1:** List of 30 national breast radiology bodies who signed a Memorandum of Understanding with the European Society of Breast Imaging and co-authored this paper

Austria	WG on Breast Imaging, Austrian Roentgen Society, Österreichische Röntgengesellschaft (ÖRG)
Belgium	Senology Section of the Belgian Society of Radiology
Bosnia and Herzegovina	Association of Radiology of Bosnia and Herzegovina
Bulgaria	Bulgarian Society of Breast Imaging
Croatia	Croatian Society of Radiology Working Group of Breast
Czech Republic	Association of Czech Breast Radiologists
Denmark	Danish Society of Breast Imaging
Estonia	Breast Imaging Subgroup of Estonian Society of Radiology
Finland	Radiological Society of Finland/Breast Radiologists of Finland
France	Société d'Imagerie de la Femme (SIFEM)
Germany	AG Mammadiagnostik / Breast Imaging Working Group of the German Roentgen Society
Greece	Hellenic Breast Imaging Society
Hungary	Section of Breast Diagnostics, Hungarian Society of Radiologists
Iceland	The Breast Imaging Group of The Radiological Society of Iceland
Ireland	Irish Breast Radiology Group
Israel	Israel Breast Imaging Society
Italy	Italian College of Breast Radiologists by SIRM (Società Italiana di Radiologia Medica)
Lithuania	Lithuanian Radiology Association
Moldova	Department of Breast Imaging in the Society of Imagists of the Republic of Moldova
The Netherlands	Dutch College of Breast Imaging (DCBI)
Norway	Norwegian Society of Breast Imaging
Poland	Sekcja Diagnostyki Obrazowej Chorób Piersi; Polskie Towarzystwo Radiologiczne
Portugal	Breast Imaging Section of Portuguese Society of Radiology and Nuclear Medicine (SPRMN)
Romania	Romanian Society of Breast Imaging
Serbia	School of Breast Imaging
Slovakia	The Section of Breast Imaging of Slovac Radiologic Society
Spain	Spanish Society of Breast Imaging, Sociedad Espaňola de Diagnostico e Interventencionismo de la Mama (SEDIM)
Sweden	Swedish Breast Imaging Society
Switzerland	Breast screening representative of the Swiss Radiological Society
Turkey	Turkish Society of Radiology Breast Imaging Working Group

## Breast cancer as a major health issue and the role of mammography in early diagnosis

All over the world, BC remains a major issue for public health. Increasing numbers of new cases and deaths are observed in both developed and less developed countries, only partially attributable to the increasing population age. In the 28 member states of the European Union, there were 361,608 new BC cases in 2012 and these are estimated to have increased to 373,733 in 2015 (+3.4 %); deaths were 91,585 and 95,357, respectively (+4.1 %) [1]. No major differences in this trend can be appreciated across European countries.

Notwithstanding its intrinsic limitations in terms of sensitivity and specificity, mammography remains the main tool for population-based mass screening with demonstrated effectiveness in reducing mortality and allowing for conservative treatment, as already stated by EUSOBI [2]. Tumour stage at diagnosis of BC still significantly impacts on overall survival even in the current era of effective systemic therapy. Thus, early diagnosis remains crucial. This principle has been recently confirmed by an interesting population-based study from the Netherlands Cancer Registry, which evaluated more than 170,000 BC patients. The proportion of patients receiving neoadjuvant/adjuvant systemic therapy increased from 53 % in 1995–2005 to 60 % in 2006–2012. However, in 2006–2012 the mortality for larger tumours remained greater than that for smaller tumours, significantly for the comparison of T1c and T1a stage, and was independent from nodal status [3].

The evidence in favour of screening mammography has been recently summarized by the International Agency for Research on Cancer (IARC) [4]. Upon randomized controlled trials, the reduction in BC mortality due to screening mammography is confirmed for women between 50 and 69 years of age. Considering 20 cohort studies and 20 case-control studies, the estimated reduction in BC mortality is 40 % for women aged 50–69 years who take up the invitation and 23 % when also including those who do not accept the invitation, as a societal effect of the screening policy. From cohort studies, a mortality reduction has also been estimated for women aged 40–49 years and 70–74 years, though the evidence from published studies was considered to be “limited”. Available data did not allow the IARC working group to define an optimal screening interval. However, we should consider that the majority of European countries opted for biennial screening in the 50- to 69-year-old cohort. When 40- to 49-year-old cohort is invited, the yearly interval is generally adopted in consideration of a potential higher speed of BC growth and of a lower sensitivity of mammography due to the higher breast density.

The average cumulative risk for a false-positive recall in organized screening programmes has been evaluated by the IARC working group to be about 20 % for women aged 50–69 years who have ten screens in 20 years, while the needle biopsy rate for a false-positive finding is lower than 1 % per round [4]. In addition, it should be noted that screening mammography allows for both downscaling clinico-pathological features of invasive BCs and reducing the impact of loco-regional and adjuvant treatments [5–8].

With regard to overdiagnosis (i.e. the rate of screen-diagnosed BCs otherwise unnoticed during the patient’s lifetime), the IARC working group accepted the estimate provided by the Euroscreen working group [9], equal to 6.5 % (range 1–10 %), which was calculated on the basis of the difference in the cumulative probability of a BC diagnosis among women receiving or not receiving screening mammography, taking into account lead time and underlying increasing incidence. If these factors are carefully considered, a similar estimate of overdiagnosis (4–11 %) is also obtained from randomized controlled trials [4]. Notably, while overdetection (resulting from a specific action by the breast radiologist evaluating a finding as suspicious) should be distinguished from overdiagnosis (which also implies an essential role of the pathologist) [10], further efforts should be dedicated to the reduction of the most important negative consequences of overdiagnosis, i.e. overtreatment.

## Risk of radiation-induced breast cancer

Radiation-induced BCs from mammography were estimated, based on models including different factors. For the 50- to 69-year age group, taking into account a latency time of 10 years and a dose of 2.5 mGy per screening round, the risk of radiation-induced BC death has been estimated to be 1 per 100,000 screened women. The risk of radiation-induced BC due to screening mammography is at least 100 times lower than the probability of avoiding a BC death [4]. Applying a mortality reduction rate of 43 %, biennial screening mammography performed in 100,000 women saves 350 lives [11]. For the 40- to 49-year age group, the problem of radiation effects must be more carefully considered and depends on the estimated magnitude of radiation- induced BCs. Importantly, most of radiation-induced BCs will be cured [12].

## Screening models

On the basis of the available evidence, the EUSOBI and the above-listed national breast radiology bodies strongly support screening mammography of the female population at average BC risk, typically from 50 to 69 years of age; extension of this up to 73–75 years, biennially, is a second priority. Extension from 40 or 45–49, with annual screening, can be evaluated as a third priority, country-by-country. Age selection and screening interval should be adapted to national demographics and local priorities. Importantly, these societies strongly discourage the use of methods for screening such as thermography or other optical imaging tools as an alternative to mammography [13]. Moreover, these societies also discourage the use of ultrasound as a primary screening tool in asymptomatic European women at average risk of BC.

Preference should be given to population-based screening programmes on a regional/national basis with double reading rather than spontaneous mammographic screening with a single reading, given the advantages of the former in terms of higher specificity and positive predictive value [14, 15], lower cost, as well as structured quality controls and central data management. This concept has also been recently reinforced by the IARC working group in the above-mentioned paper [4].

In a wider framework, the EUSOBI and the above-listed national breast radiology bodies are aware of the open debate in other contexts such as that in the USA where the Society of Breast Imaging and the American College of Radiology support annual screening mammography from the age of 40 years by informing women on the advantages of early BC diagnosis [16]. The recent recommendations of the American Cancer Society [17] can be a reference for the US context: (1) regular screening mammography starting at age 45 years (strong recommendation); (2) annual screening mammography from 45–54 years of age (qualified recommendation); (3) from 55 years of age, transition to biennial or continuing annually (qualified recommendation); (4) opportunity to begin annual screening from 40–44 years (qualified recommendation); (6) continue screening mammography as long as women’s overall health is good and they have a life expectancy of ≥10 years (qualified recommendation); (7) no suggestion for screening clinical breast examination at any age (qualified recommendation).

## Breast density

The EUSOBI and the above-listed national breast radiology bodies are aware of the masking effect of increased breast density, strongly impacting on the sensitivity of screening mammography, declining from 86–89 % for almost entirely fatty breasts to only 62–68 % for extremely dense breasts [18]. Studies aimed at reducing this negative effect by means of supplemental screening tools, such as manual or automated breast ultrasound, are welcome, especially when evaluating the cost-effectiveness of the additional tools on the large scale of population-based screening programmes. These societies also take into consideration the role of breast density as an independent BC risk factor, although this factor can be over-rated [19, 20], especially when reported as a communication to the women. In studies with a control group not limited to fatty breasts, the relative risk of women with dense breasts dropped to 2 or less [21, 22]. At any rate, these societies consider the general adoption of direct digital mammography to be the first priority to improve the sensitivity in women with increased breast density.

## The potential of digital breast tomosynthesis

These societies also consider the increasing evidence in favour of digital breast tomosynthesis (DBT) as a screening tool. Three prospective studies showed that DBT used as an adjunct [23–25] or alternative [26] to two-dimensional (2D) digital mammography allows for a superior diagnostic performance when compared to the latter alone. Overall, DBT increases the detection rate from 0.5 to 2.7 per 1,000 screened women and reduces the recall rate from 0.8 to 3.6 per 100 screened women [27]. Of note, DBT is now proposed along with synthetic 2D views, practically solving the problem of an increased radiation exposure when DBT is performed as an adjunct to 2D digital mammography [28–30]. All these aspects will probably also confer to DBT the status of future “routine mammography” in the screening setting. However, before introducing DBT in BC screening outside trials approved by ethical committees, we need evidence for a statistically significant and clinically relevant reduction in the interval cancer rate. This cautiousness is due to the need to avoid an increase in overdiagnosis and costs, in the absence of the demonstration of cost-effectiveness of screening DBT (proof of which may require very long studies). First results on a reduction from 0.7 to 0.5 interval cancers per 100 screened women were very recently reported from a large study in the USA [31], but further evidence is needed. Moreover, the probable increase in reading time associated with the use of DBT in screening [32] and its effects on sustainability of screening programmes should be considered before routine implementation.

## Preference for digital instead of film-screen mammography

Overall, looking at the course of technological evolution of mammography in the last decades and at the current trend in favour of DBT, these societies strongly support the adoption of direct digital mammography (not phosphor-plate computer radiography) instead of film-screen mammography in all countries. In fact, digital mammography implies many substantial advantages, including lower dose, higher image quality, possibility of post-processing, digital archiving, image transmission and no chemical pollution. We suggest that new mammographic units should be based on direct digital mammography technology and, when possible, equipped with DBT in readiness for the next evolution.

## Need for certified and subspecialty-trained radiologists in the context of breast centres

Screening mammograms, with or without DBT, should be read by radiologists qualified as screening mammography readers. Proficiency tests at the regional/national/European level are encouraged in order to guarantee a standardized reading quality together with minimum screening numbers read per year.

It is essential that there is a continuity of care from screening mammography to diagnostic breast imaging, to needle sampling and treatment planning either in the context of a dedicated breast centre or in a screening centre that has a well-organized relationship with a diagnostic imaging facility. Whenever possible, radiologists should operate in the context of integrated breast units with the help of organized/structured cooperation among BC specialists.

Quality assurance programmes regarding breast radiology units/sections are also encouraged in the context of forthcoming new European guidelines for BC screening, diagnosis and treatment.

## Preference for core or vacuum-assisted biopsy

Preference should be given to needle sampling of breast lesions using core biopsy or vacuum-assisted biopsy instead of fine needle aspiration [33], in consideration of the lower false-negative rate and/or inadequate sampling, unless strict cooperation with a cytologist allows for a demonstrable equally high diagnostic performance. This preference does not apply for sampling of lymph nodes suspected to be metastatic at ultrasound of axilla, where fine needle aspiration has been shown to be effective [34].

## Women at increased risk for breast cancer

These societies are in favour of including, whenever possible, dedicated pathways for high-risk women (lifetime risk equal to or higher than 20 %), offering magnetic resonance imaging according to national or international guidelines and recommendations [35–37]. In this regard, policies will be different, considering the heterogeneity of health systems across countries. Studies considering risk stratification for different screening strategies of women at increased BC risk are welcome.

## Summary statement

EUSOBI and 30 national breast radiology bodies strongly support mammography as a population-based mass screening tool which results in a relevant reduction in BC mortality and leads to a favourable decrease in both loco-regional and adjuvant treatments in women attending these programmes. People and institutions questioning its validity despite a large body of evidence accumulated in more than three decades put women’s lives at risk.
